# The Use of Awake Videolaryngoscopy and an Innovative Flexible-Tipped Bougie in a Potentially Difficult Airway

**DOI:** 10.1155/2020/8850665

**Published:** 2020-07-04

**Authors:** Will A. Watson, John Cormack

**Affiliations:** ^1^Anesthetic Fellow, St Vincent's Hospital, Melbourne, Australia; ^2^Consultant Anesthetist, St Vincent's Hospital, Melbourne, Australia

## Abstract

We present a case where awake videolaryngoscopy (VL) was used, along with a flexible-tipped bougie to allow endotracheal intubation in a challenging airway. The patient presented for resection of a large left-sided parapharyngeal mass. Examination and imaging led to concerns about a potentially difficult airway. Awake VL was used to assess the grade of intubation before induction of anesthesia. Once the patient was anesthetized, a flexible-tipped bougie was used to navigate past the mass, allowing successful intubation. This case report demonstrates the combination of these two technologies to provide effective airway management in the potentially difficult airway.

## 1. Introduction

We present a case involving a potentially difficult airway due to a pharyngeal mass, where awake videolaryngoscopy and a flexible-tipped bougie were utilized to ensure the safe passage of an endotracheal tube without complication or airway trauma. This case report is published with the written consent of the patient. No ethical approval was required for publication.

## 2. Case Description

**Figure 1:**
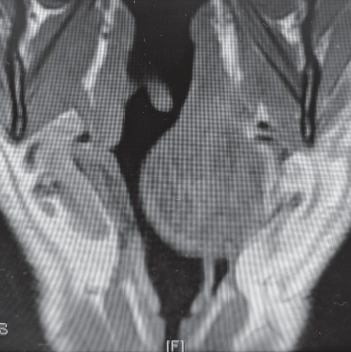
View of mass on axial CT.

A 61-year-old man was referred to the ear, nose, and throat (ENT) service with an 8-year history of worsening sleep apnea (the Apnea-Hypopnea Index 65). The initial diagnosis was made following a sleep study and treated with home continuous positive airway pressure (CPAP), but increasing levels of positive airway pressure were required and the development of a left-sided neck lump prompted further investigation and imaging. Magnetic resonance imaging demonstrated a submucosal mass measuring 5.8 cm by 4.2 cm, bulging anteriorly and to the left, occupying most of the hypopharynx above the level of the false vocal cords (Figure 1). Outpatient nasendoscopy by the ENT surgeon was noted to provide a poor view of the larynx due to the pharyngeal mass.

He was otherwise fit and well, with a past medical history of ischemic heart disease. He was an ex-smoker, with an exercise tolerance of >4 metabolic equivalent of tasks (METs). He had a body mass index of 34 (height: 184 cm; weight: 115 kg). Preoperative airway assessment revealed interincisal distance greater than 3 cm, slightly limited neck extension, and was a Mallampati grade 3. The patient was connected to full monitoring as per The Association of Anaesthetists of Great Britain and Ireland standards. He was given 200 mcg of glycopyrrolate intravenously for secretion suppression. Midazolam 0.5 mg was administered intravenously for anxiolysis, and a remifentanil target-controlled infusion was commenced at 2 ng/ml and titrated to effect up to 4 ng/ml. The airway was topicalized with 6 mls of 4% lidocaine applied with the MADgic laryngo-tracheal mucosal atomization device (Teleflex medical, Carrington, NC, USA). The dose was divided between the pharynx and base of the tongue, the hypopharynx, and the supraglottis, inserted blind and timed with inspiration.

**Figure 2:**
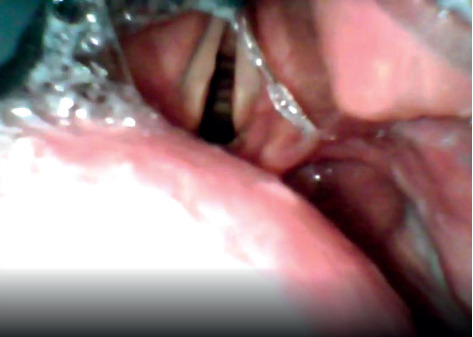
View on awake videolaryngoscopy.

A GildeScope 3 LoPro (Verathon, Bothell, United States of America) was inserted after topicalisation, to assess the airway (Figure 2).

**Figure 3:**
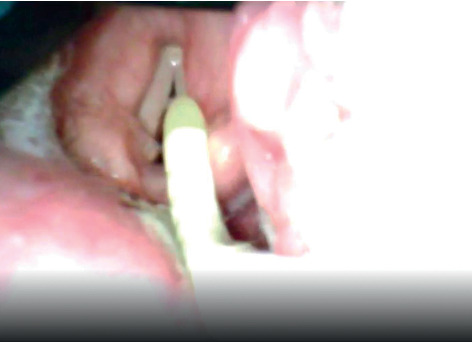
Flexible-tipped bougie in flexed position.

**Figure 4:**
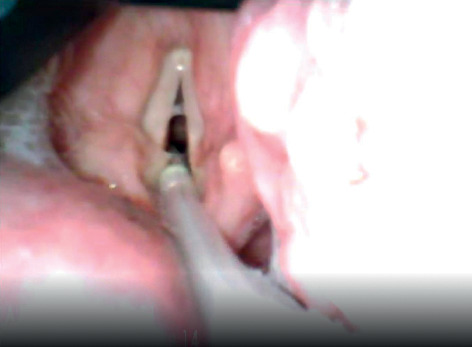
Flexible-tipped bougie in neutral position.

Deep insertion of the videolaryngoscope over the epiglottis was well tolerated by the patient and glottic exposure was allowed, despite the tumor bulging into the field of view. An assessment that intubation using a videolaryngoscope would likely be successful under general anesthetic was made. The patient was subsequently anesthetized with propofol 200 mg and rocuronium 70 mg, whilst maintaining the remifentanil at a target concentration of 4 ng/ml. The GildeScope was inserted again after confirmation of paralysis, and a view of the cords obtained. A flexible-tipped Bougie (Construct medical, Melbourne, Victoria) was manipulated over the tumor and through the vocal cords at the first attempt (Figures 3 and 4). See Supplementary Video 1, for the footage of the intubation using the flexible-tipped bougie.

The airway was secured with a size 7.5 endotracheal tube, railroaded over the bougie, and the surgery proceeded successfully with maintenance anesthesia provided by sevoflurane in air and oxygen. He was given paracetamol 1 g, ondansetron 4 mg, dexamethasone 8 mg, and fentanyl 100 mcg intraoperatively, and a phenylephrine infusion was used to maintain his blood pressure at acceptable levels.

He made an uneventful recovery from anesthesia. Postoperatively, the patient's obstructive sleep apnea symptoms had almost completely resolved, and he no longer required overnight CPAP. Histology confirmed the tumor as a low-grade sarcoma. The patient was referred on for radiotherapy.

## 3. Discussion

This case provides a good example of the challenges involved in planning the management of a potentially difficult airway and also demonstrates how recent technological advances have allowed better real-time assessment and management of cases, which would have previously been managed either with an awake fibreoptic intubation and elective front of neck access or with general anesthesia and crossed fingers. The use of awake videolaryngoscopy for preoperative assessment has been well described [1], and in this case, it provided reassurance that an acceptable view of the vocal cords would be obtainable. Thus, a hypnotic agent and muscle relaxant could be given, with confidence. The flexible-tipped bougie was easily maneuvered around the mass and through the vocal cords, avoiding the commonly encountered issues with challenging bougie manipulation anteriorly through the cords and subsequent holdup subglottically.

A difficult airway manikin study compared videolaryngoscope intubation using a FROVA intubating introducer (Cook Medical, Bloomington, IN, USA) with the Flexible Tip Bougie used in our case. It is found that the Flexible Tip Bougie provided a faster time to intubation and a greater ease of use than the FROVA [2]. A recent clinical trial with expected difficult intubations and a videolaryngoscope plus a flexible bronchoscope being used as a tube introducer demonstrated superiority with respect to first pass success, time to intubation, and airway trauma compared with a videolaryngoscope using a preshaped nondynamic stylet-guided endotracheal tube [3]. We feel that our patient may have been more difficult to intubate with a stylet-guided endotracheal tube as the available glottic visibility was significantly reduced by the large tumor. The much smaller diameter of the flexible-tipped bougie and the ability to flex into the laryngeal inlet and then extend down the trachea made a potentially difficult intubation seem straightforward.

This case report adds to the literature supporting the use of awake VL for assessment of patients with potentially difficult airways due to upper airway masses. It also further supports the effectiveness of the flexible-tipped bougie in combination with VL to allow the atraumatic passing of an endotracheal tube, as has previously been reported [4]. The flexible-tipped bougie allows control of the device tip to the cords and then, subglottically, as a viable alternative to using an introducer stylet when intubating patients using VL.

## Data Availability

No data were used to support this study.

## Abbreviations

VL: Videolaryngoscopy
ENT: Ear, nose, and throat
CPAP: Continuous positive airway pressure
MET: Metabolic equivalent of task.

## Supplementary Materials

Supplementary Materials A video showing the mass on CT, initial look with videolaryngoscopy under sedation, subsequent intubation using flexible-tipped bougie, and demonstration of the function of the flexible-tipped bougie.
